# Lower risk of ischemic stroke among patients with chronic kidney disease using chinese herbal medicine as add-on therapy: A real-world nationwide cohort study

**DOI:** 10.3389/fphar.2022.883148

**Published:** 2022-08-11

**Authors:** Hsuan-Shu Shen, Chung-Yi Hsu, Hei-Tung Yip, I-Hsin Lin

**Affiliations:** ^1^ Department of Chinese Medicine, Hualien Tzu Chi Hospital, Buddhist Tzu Chi Medical Foundation, Hualien, Taiwan; ^2^ School of Post-Baccalaureate Chinese Medicine, Tzu Chi University, Hualien, Taiwan; ^3^ Sports Medicine Center, Hualien Tzu Chi Hospital, Buddhist Tzu Chi Medical Foundation, Hualien, Taiwan; ^4^ Institute of Medical Sciences, Tzu Chi University, Hualien, Taiwan; ^5^ Graduate Institute of Biomedical Sciences, China Medical University, Taichung, Taiwan; ^6^ Management Office for Health Data, China Medical University Hospital, Taichung, Taiwan; ^7^ College of Medicine, China Medical University, Taichung, Taiwan

**Keywords:** chronic kidney disease, ischemic stroke (IS), Chinese herbal medicine, inflammation, traditional Chinese medecine

## Abstract

**Background:** The incidence of ischemic stroke (IS) is much higher among patients with chronic kidney disease (CKD) compared to the general population. Few studies have evaluated the association between the risk of IS and the use of Chinese herbal medicine (CHM) in patients with CKD. We aimed to investigate the risk of IS among patients with CKD using CHM as add-on therapy.

**Methods:** We conducted a retrospective cohort study based on Taiwan’s National Health Insurance Research Database to assess 21,641 patients with newly diagnosed CKD between 2003 and 2012. Patients were classified as either the CHM (n = 3,149) or the non-CHM group (*n* = 3,149) based on whether they used CHM after first diagnosis of CKD. We used the proportional subdistribution hazards model of Fine and Gray to examine the hazard ratio (HR) of IS in propensity-score matched samples at a ratio of 1:1 for two groups.

**Results:** The risk of IS was significantly reduced in the CHM group (adjusted HR [aHR]: 0.58, 95% confidence interval [CI]: 0.48–0.70) compared with the non-CHM group. Those who used CHM for >180 days had an even lower risk of IS than those in the non-CHM group (aHR: 0.51, 95% CI: 0.41–0.63). Additionally, frequently prescribed formulae, such as Ji-Sheng-Shen-Qi-Wan, Liu-Wei-Di-Huang-Wan, and Zhen-Wu-Tang were associated with a 30%–50% reduced risk of IS.

**Conclusion:** Our results suggest that patients with CKD who used CHM as add-on therapy had a lower hazard of IS than those in the non-CHM group, especially for patients taking CHM for >180 days. Further experimental studies are required to clarify the causal relationship.

## Background

Chronic kidney disease (CKD) has recently been reported as an independent risk factor for stroke. The incidence of ischemic stroke (IS) was much higher among patients with CKD compared to the general population (Tsagalis et al., 2009). A stroke in patients with CKD was associated with a worse functional outcome, a 138% higher risk of in-hospital mortality, a 49% higher risk of neurological deterioration during their hospitalization and greater medical complications including neurological deterioration, urinary tract infections, respiratory infections, and pressure sores than general population (Grams et al., 2017; Kelly and Rothwell, 2020; Kelly et al., 2021). Additionally, the IS in the advanced CKD (stage 4 or 5) patients is associated with an even higher mortality rate and higher risk of kidney failure (Wetmore et al., 2020). Abramson et al. conducted a prospective study (Atherosclerosis Risk in Communities study) which revealed that the risk of stroke was almost doubled in patients with CKD after controlling for conventional cardiovascular risk factors (Abramson et al., 2003). Furthermore, according to a systematic review study, the relative risk of IS was 1.63 (confidence interval [CI]:1.34–1.99) after adjusting the traditional risk factors of ischemic stroke (Kelly and Rothwell, 2019). The mechanism of stroke might be associated with endothelial dysfunction and accelerated arteriosclerosis (Chelluboina and Vemuganti, 2019). The accumulation of uremic toxins due to the gradual decline of kidney function aggravated the endothelial dysfunction (Jing et al., 2018). Indoxyl sulfate, one of the uremic toxins, induced the production of reactive oxygen species, promoting the pathways of activating protein 1 and nuclear factor Kappa B (NF-κB). These pathways are known to be associated with inflammation (Lano et al., 2020). In addition, renin-angiotensin system-induced angiotensin-2 activated the inflammatory reaction and induced vascular fibrosis (Benigni et al., 2010). This kind of oxidative stress response exacerbated arteriosclerosis and arterial stiffness and consequently contributed to IS among patients with CKD (Deanfield et al., 2007; Ma et al., 2015). Since the complications of CKD such as stroke increased mortality and reduced health related quality of life, it was important to slow its progression and reduce the potential complications.

The primary medications for preventing stroke among patients with CKD included antiplatelets, anticoagulants, statins, and antihypertensive agents (Kelly and Rothwell, 2020). These conventional medications have been shown to have a protective effect against stroke in the general population. However, certain kinds of antiplatelets and anticoagulants, such as aspirin, clopidogrel, and warfarin, might contribute to an increased risk of bleeding among patients with CKD (Olesen et al., 2012; Palmer et al., 2013). Antihypertensive agents, such as angiotensin-converting enzyme inhibitors and angiotensin receptor blockers, could slow the progression of kidney disease, but robust evidence regarding stroke prevention was still lacking (Kelly and Rothwell, 2020). There have been limited evidence for strategies to prevent stroke in patients with CKD and more studies on novel therapeutic interventions are needed (Levin et al., 2017).

In addition to conventional medicine, 25%–50% of CKD patients used complementary and alternative medicine (CAM). The main reason for CAM usage was based on the potential benefits of CAM (Lin et al., 2015; Osman et al., 2015). The potential benefits of CAM for patients with CKD include prolonging time of progression to kidney failure as well as alleviating association problems, including arthritis, pruritus, cardiovascular risk factors, anxiety, depression, and fatigue (Markell, 2005). Among CAM, herbal products were the most commonly used type (Akyol et al., 2011). Based on previous studies, using Chinese herbal medicine (CHM) as add-on therapy with conventional therapy provided better renal protective effects in CKD patients than receiving conventional therapy alone. Patients treated with *Abelmoschus manihot* (L.) Medik [Malvaceae; Abelmoschi Corolla (Huang-Shu-Kui)] plus lorsatan for 24 weeks had less proteinuria than those treated with lorsatan alone (Zhang et al., 2014). In addition, patients with CKD using Dang-Gui-Bu-Xue-Tang or Fang-Ji-Huang- Qi-Tang and Benazepril in combination had higher estimated glomerular filtration rates (eGFR) and lower proteinuria than those using Benazepril alone (Wang Y. J. et al., 2012). Moreover, a meta-analysis of clinical trials also demonstrated that adding Liu-Wei-Di-Huang-Wan to western medicine might improve renal function including lowering the level of blood urea nitrogen (BUN), improving 24-hour urine total protein, and decreasing the level of serum creatinine (Lin et al., 2016). Furthermore, the underlying mechanism of CHM exerting beneficial effects in patients with CKD have been investigated through animal models. Liu-Wei-Di-Huang-Wan was found to reduce the ameliorate oxidative stress response in diabetic nephropathy rats (Xu et al., 2017). Additionally, Liu-Wei-Di-Huang-Wan was shown to have anti-inflammatory effects through preventing the activation of NF-κB signaling pathways (Xu et al., 2017). Given that some CHM have been suggested to have renal protective and anti-inflammatory effects, we hypothesized that CHM as add-on therapy with conventional therapy may be associated with a decreased risk of IS among patients with CKD. Accordingly, we conducted a retrospective cohort study to evaluate the risk of IS among patients with CKD using a combination of CHM and conventional medicine.

## Methods

### Data resource

Taiwan launched a single-payer National Health Insurance (NHI) Program in 1995 that coveres almost the entire population. The annual coverage rate has ranged from 96.1% to 99.6% of the population, with more than 20 million Taiwan residents enrolled since 1997. The National Health Insurance Research Database (NHIRD) derived from the NHI program has been used for research purposes. The data we used in this study was from a longitudinal health insurance database from 2002 to 2013 comprised of medical claim data of one million beneficiaries randomly selected from the NHIRD, representing about 4.2% of all enrollees. Data in 2002 were used to identified patients’ medication history and comorbidities. Data during 2003–2012 were used to identify cases with newly diagnosed CKD. Data in 2013 were used for the subsequent follow-up. We extracted data regarding demographic characteristics, including encrypted identification numbers, gender, date of birth and death, and diagnostic information. The diagnostic data contained International Classification of Disease, Ninth Revision, Clinical Modification (ICD-9-CM) as well as diagnostic and procedure codes. This study complied with the Declaration of Helsinki and was approved by the Institutional Review Board of the China Medical University (CMUH104-REC2–115 [CR-6]).

### Study population

The study cohort was composed of the patients with a diagnosis of CKD (ICD-9-CM codes 585 and 586) and having at least three outpatient visits or one inpatient visit within 1 year from 2003 to 2012. The cohort was divided into two groups: those using CHM because of CKD diagnosis (prescribed CHM accompanied with CKD diagnosis code, ICD-9-CM codes 585 and 586) during the study period were allocated to the CHM group; those who did not receive any CHM treatment during the study period were allocated to the non-CHM group. The index date for the CHM group was the first date of taking CHM because of the CKD diagnosis, and the index date of the non-CHM group was randomly assigned a date between CKD diagnosis date and 31 December 2013. We excluded patients who were under 18 years of age, experienced a stroke (ischemic stroke or hemorrhage stroke) before the index date, had dialysis history before the index date (possible history of CKD), and had IS occurring during the first 6 months after CKD diagnosis date. Additionally, we conducted propensity score matching at a ratio of 1:1 cohort sample by age (per 5 years-groups), gender, index year, comorbidities and medication to balance the basic characteristics of two groups.

### Outcome, comorbidity and medication

The main outcome of the study was IS (ICD-9-CM codes 433–437). We followed patients until IS occurred, death, loss to follow-up or 31 December 2013. Death was considered as the competing event; loss to follow-up and the end of this study were considered as censored events.

Baseline comorbidity was defined as comorbid disease with at least three outpatient visits or at least 1 inpatient visit before the index date within 1 year. According to the recommendation of the previous study, hypertension (ICD-9-CM codes 401–405), diabetes mellitus (ICD-9-CM code 250), hyperlipidemia (ICD-9-CM code 272), and atrial fibrillation (ICD-9-CM code 427.31) are thought to be the traditional risk factors for patients with CKD (Kelly and Rothwell, 2020). In addition, chronic obstruction pulmonary disease (COPD, ICD-9-CM codes 491, 492, and 496), chronic liver disease and cirrhosis (ICD-9-CM code 571), coronary artery disease (ICD-9-CM codes 410–414), and cancer (ICD-9-CM codes 140–280) were also included as the covariates. We considered the possible effect of medications for risk of IS as follows: diabetic medications, antihypertensive medications, anti-lipid medications, non-steroidal anti-inflammatory drugs (NSAIDs), steroid, and antiplatelet or anticoagulation agents. Furthermore, a sensitivity analysis was conducted to assess the robustness of the findings. Originally, we excluded patients who had IS occurring during the first 6 months after CKD diagnosis date. For the sensitivity analysis, we extended this period of case exclusion to 12 months and evaluated the impact of longer induction time on the incidence rate of IS between two groups.

### Statistical analysis

Continuous variables are reported as mean and standard deviation, whereas categorical variables are reported as frequencies and percentage. We used standardized mean difference to compare balance of basic characteristics and potential confounders between the two groups, and a value of <0.1 indicates a negligible difference between the two groups. Considering the presence of competing risk, we used the proportional subdistribution hazards model of Fine and Gray to examine the effect of CHM on the risk of IS, reporting as hazard ratio (HR) and 95% confidence interval (CI). A multivariable model was performed by controlling for potential confounding variables, such as gender, age, comorbidities of hypertension, diabetes mellitus, hyperlipidemia, atrial fibrillation, COPD, chronic liver disease and cirrhosis, chronic artery disease, cancer, and the use of diabetes medications, antihypertensive medications, anti-lipid medications, steroid, NSAIDs, and antiplatelet or anticoagulation agents. We applied cumulative incidence function to assess the cumulative incidence rate of IS between the two groups. The Gray’s test (Williamson et al., 2007) was used to compare the differences between the two groups. In addition, subsequent subgroup analysis was performed to examine the effect of cumulative CHM usage days. We stratifised the patients in the CHM group into three subgroups: cumulative CHM usage days ≤90 days, 91–180 days, and >180 days. All statistics significances were set at a *p* < 0.05 and the SAS 9.4 (SAS Institute Inc. Cary, NC) was used for the statistical analysis.

## Results

**FIGURE 1:**
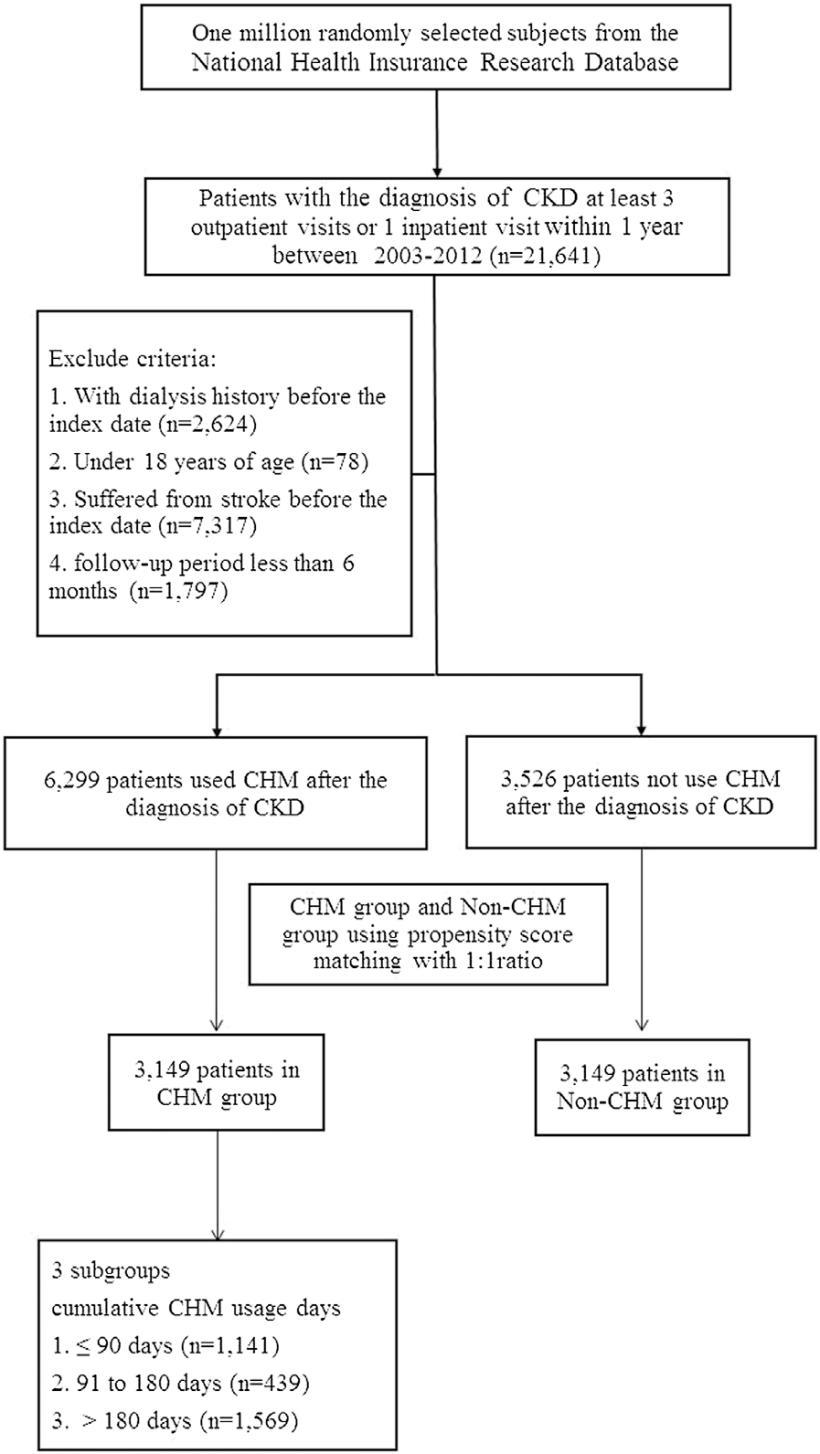
Study flowchart of patients with chronic kidney disease. After excluding patients not fitting inclusion criteria, CHM and non-CHM groups comprised 3,732 patients at a ratio of 1:1 cohort sample for propensity score matching.

We identified 21,641 patients diagnosed with CKD during the years 2003–2012. According to the exclusion criteria, 11,816 patients were excluded. A total of 6,299 patients received CHM because of CKD diagnosis, and 3,529 patients received conventional treatment only. After matching with propensity score at a ratio of 1:1 cohort sample by age (per 5 years-groups), gender, index year, comorbidities and medication, there were 3,149 patients in either the CHM group or the non-CHM group. The sampling procedure is presented in Figure 1.

Table 1 shows the demographic characteristics, comorbidities, and confounding medications of the study patients. There was no difference between the two groups with regard to gender, age comorbidities, and medications (standardized mean difference <0.1). The mean follow-up period of the CHM group was longer than that of the non-CHM group [3.96 (2.78) years vs. 2.90 (2.35) years; standardized mean difference = 0.412]. The period from index date to IS date was also longer in CHM group than that in non-CHM group [3.13 (2.37) years vs. 2.28 (1.73) years; standardized mean difference = 0.407].

**TABLE 1:** Characteristics of patients with chronic kidney disease classified according to the use of Chinese herbal medicine after matching with propensity score.

	Non-CHM group		CHM group		Standardized difference^§^
	(n = 3,149)		(n = 3,149)		
	n	%	n	%	
Gender					0.020
Female	1,154	36.6	1,124	35.7	
Male	1,995	63.4	2,025	64.3	
Age					
<50	444	14.1	344	10.9	0.096
50–64	860	27.3	953	30.3	0.065
≥65	1,845	58.6	1,852	58.8	0.005
mean (SD)	66.6	(15.1)	66.41	(12.8)	0.014
Comorbidity					
Hypertension	1,637	52.0	1,646	52.3	0.006
Diabetes mellitus	969	30.8	953	30.3	0.011
Hyperlipidemia	612	19.4	597	19.0	0.012
Atrial fibrillation	57	1.8	62	2.0	0.012
COPD	210	6.7	201	6.4	0.012
Chronic liver disease and cirrhosis	236	7.5	234	7.4	0.002
Coronary artery disease	77	2.4	70	2.2	0.015
Cancer	272	8.6	271	8.6	0.001
Medication					
Insulin Analogues	768	24.4	773	24.5	0.004
Oral hypoglycemic agent	1,104	35.1	1,098	34.9	0.004
ARB/ACEI	2,042	64.8	2,065	65.6	0.015
Antihypertensive agent except ARB/ACEI	2,930	93.0	2,943	93.5	0.016
NSAIDs	3,083	97.9	3,080	97.8	0.007
Analgesic drugs except NSAIDs	3,124	99.2	3,121	99.1	0.010
Anti-lipid drug	1,445	45.9	1,436	45.6	0.006
Steroid	2,677	85.0	2,704	85.9	0.024
Antiplatelet or anticoagulation agent	2,157	68.5	2,169	68.9	0.008
Period from diagnosis date to index date, year [mean (SD)]	0.22	(0.25)	0.17	(0.24)	0.191
Follow-up period, year [mean (SD)]	2.90	(2.35)	3.96	(2.78)	0.412
Period from index date to ischemic stroke date, year [mean (SD)]	2.28	(1.73)	3.13	(2.37)	0.407

CKD, chronic kidney disease; CHM, Chinese herbal medicine; SD, standard deviation; COPD, chronic obstructive pulmonary disease; ARB, angiotensin receptor blocker; ACEI, angiotensin-converting enzyme inhibitors; NSAIDs, nonsteroidal anti-inflammatory drugs.

^§^A standardized mean difference of ≤0.10 indicates a negligible difference between the two cohorts

**FIGURE 2:**
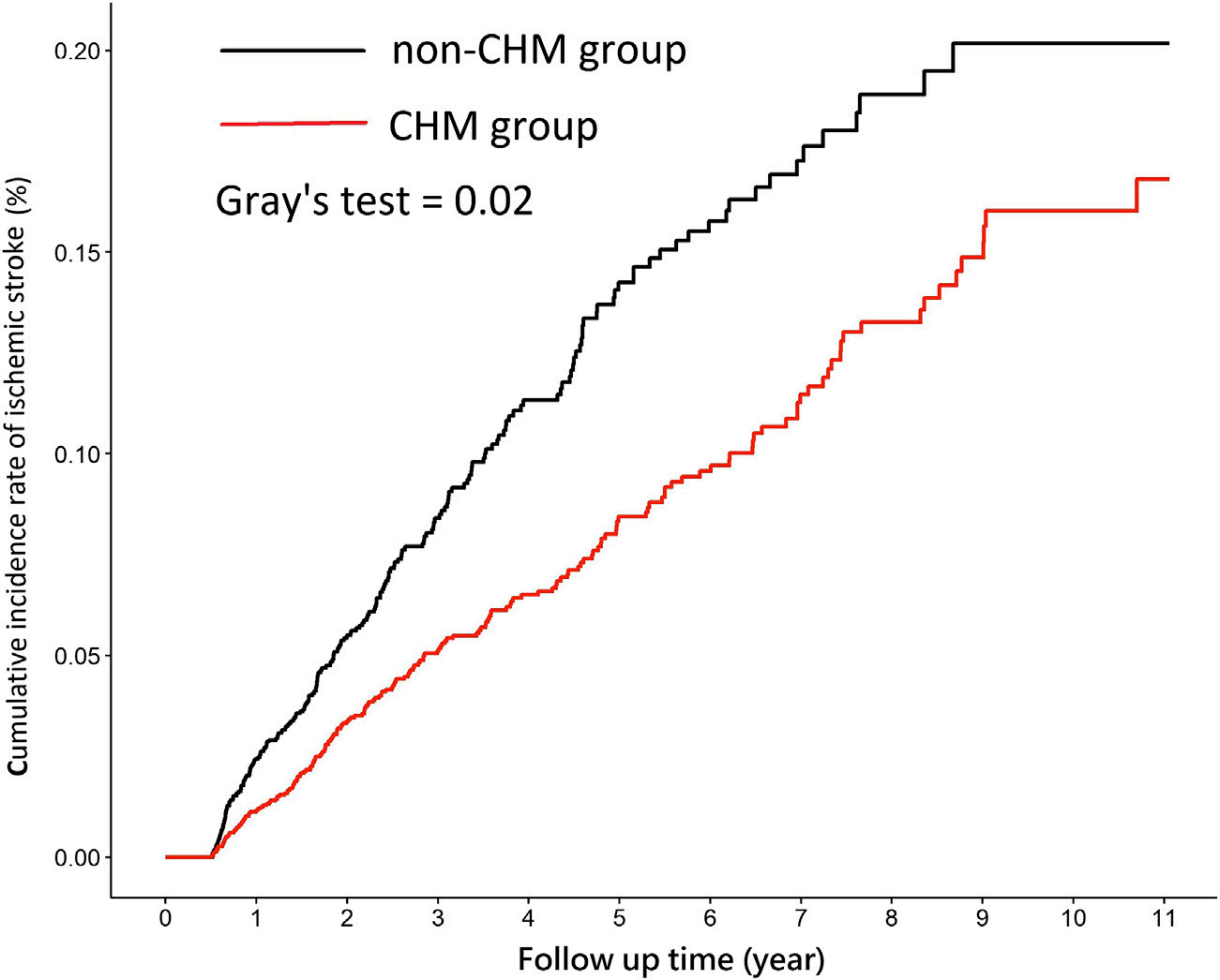
Cumulative incidence rate of ischemic stroke among patients with CKD in the CHM and non-CHM group. Abbreviate: CKD, chronic kidney disease; CHM, Chinese herbal medicine.

Overall, a total of 434 patients developed IS during the follow-up period; the incidence rates of IS in the CHM and non-CHM group was 16.05 per 1000 person-years and 25.65 per 1000 person-years, respectively (Table 2). As shown in Figure 2, the cumulative incidence function demonstrated that the cumulative incidence of IS in the CHM group was significantly lower than that in the non-CHM group (Gray’s test, *p* = 0.02). Additionally, the risk of IS was significantly lower in the CHM group than that in the non-CHM group (crude HR: 0.62, 95% CI: 0.51–0.75). After multivariate adjustment, the adjusted HR (aHR) for the risk of IS among patients with CKD taking CHM as add-on therapy remained significantly lower (aHR: 0.58, 95% CI: 0.48–0.70). After stratification by gender, both male and female patients with CKD treated with CHM were significantly associated with a reduced risk of IS, and the risk of IS was much lower in female patients (aHR: 0.49, 95% CI: 0.35–0.69) than that in male patients (aHR: 0.63, 95% CI: 0.50–0.80). Furthermore, patients with CKD aged ≥65 years who received CHM had significantly lower IS risk compared with patients of the same age not receiving CHM (aHR: 0.55, 95% CI: 0.44–0.69). Regarding comorbidities, CKD patients with the traditional IS risk factors, such as hypertension, diabetes mellitus, and hyperlipidemia receiving CHM treatment were associated with a 45%–52% reduced risk of IS compared to those not treated with CHM (aHR: 0.48, 0.53, 0.55, respectively). Moreover, in the stratification analysis of the medication, CKD patients receiving the CHM in combination with conventional medications showed a trend of reduced risk of IS compared with those receiving conventional medications only (Table 2).

**TABLE 2:** Incidence rates, hazard ratio and confidence intervals of ischemic stroke among chronic kidney disease patients with and without Chinese herbal medicine usage in the stratification of gender, age, comorbidities and medication.

Variables	Non-CHM group (n = 3,149)			CHM group (n = 3,149)			Crude HR (95%CI)	Adjusted HR (95%CI)
	Event	PY	Ir	Event	PY	Ir		
Overall								
Ischemic stroke	234	9122	25.65	200	12,460	16.05	0.62 (0.51,0.75)***	0.58 (0.48,0.70)***
Gender								
Female	87	3116	27.92	58	4484	12.93	0.46 (0.33, 0.64)***	0.49 (0.35, 0.69)***
Male	147	6006	24.48	142	7976	17.80	0.72 (0.57, 0.91)**	0.63 (0.50, 0.80)***
Age								
≤49	11	1648	6.67	9	1667	5.40	0.8 (0.33, 1.95)	0.51 (0.18, 1.39)
50–64	47	2522	18.64	52	3962	13.12	0.69 (0.46, 1.03)	0.67 (0.45, 1.01)
≥65	176	4952	35.54	139	6831	20.35	0.56 (0.45, 0.7)***	0.55 (0.44, 0.69)***
Comorbidity								
Hypertension								
No	94	4750.9	19.79	98	6420	15.26	0.78 (0.58, 1.03)	0.73 (0.54, 0.97)*
Yes	140	4371	32.03	102	6040	16.89	0.51 (0.39, 0.66)***	0.48 (0.37, 0.63)***
Diabetes mellitus								
No	153	6729	22.74	133	9183	14.48	0.64 (0.51, 0.81)***	0.60 (0.48, 0.76)***
Yes	81	2393	33.84	67	3277	20.45	0.56 (0.41, 0.78)***	0.53 (0.38, 0.73)***
Hyperlipidemia								
No	194	7631	25.42	166	10,556	15.73	0.62 (0.5, 0.76)***	0.59 (0.48, 0.72)***
Yes	40	1491	26.83	34	1904	17.86	0.63 (0.4, 1.01)	0.55 (0.34, 0.88)*
Atrial fibrillation								
No	227	9020	25.17	192	12,263	15.66	0.62 (0.51, 0.75)***	0.58 (0.48, 0.70)***
Yes	7	102	68.75	8	197	40.57	0.59 (0.2, 1.73)	0.58 (0.17, 1.95)
COPD								
No	218	8612	25.31	178	11,658	15.27	0.6 (0.49, 0.73)***	0.56 (0.46, 0.69)***
Yes	16	510	31.34	22	802	27.44	0.81 (0.42, 1.56)	0.73 (0.36, 1.47)
Chronic liver disease and cirrhosis								
No	218	8459	25.77	183	11,511	15.90	0.61 (0.5, 0.75)***	0.58 (0.47, 0.7)***
Yes	16	663	24.14	17	949	17.91	0.73 (0.36, 1.44)	0.6 (0.28, 1.26)
Coronary artery disease								
No	223	8952	24.91	194	12,222	15.87	0.63 (0.52, 0.77)***	0.59 (0.49, 0.72)***
Yes	11	171	64.51	6	238	25.20	0.4 (0.15, 1.11)	0.44 (0.13, 1.46)
Cancer								
No	214	8439	25.36	182	11,568	15.73	0.62 (0.5, 0.75)***	0.58 (0.47, 0.7)***
Yes	20	683	29.27	18	892	20.17	0.67 (0.35, 1.26)	0.58 (0.3, 1.13)
Medications								
Insulin Analogues								
No	168	7215	23.29	141	9775	14.42	0.62 (0.5, 0.78)***	0.59 (0.47, 0.74)***
Yes	66	1907.5	34.6	59	2685	21.97	0.57 (0.4, 0.82)**	0.55 (0.38, 0.79)**
Oral hypoglycemic agent								
No	148	6321	23.41	119	8638	13.78	0.6 (0.47, 0.76)***	0.56 (0.43, 0.71)***
Yes	86	2801	30.70	81	3822	21.20	0.64 (0.47, 0.86)**	0.61 (0.44, 0.83)**
ARB/ACEI								
No	64	3497	18.30	64	4647	13.77	0.74 (0.52, 1.05)	0.73 (0.51, 1.04)
Yes	170	5625	30.22	136	7813	17.41	0.57 (0.45, 0.71)***	0.53 (0.43, 0.67)***
Antihypertensive agent except ARB/ACEI								
No	7	891	7.86	9	1010	8.91	1.14 (0.42, 3.06)	0.67 (0.21, 2.12)
Yes	227	8231	27.58	191	11,450	16.68	0.59 (0.49, 0.72)***	0.57 (0.47, 0.69)***
NSAID								
No	6	248	24.21	7	370	18.90	0.81 (0.27, 2.46)	0.58 (0.13, 2.58)
Yes	228	8874	25.69	193	12,090	15.96	0.61 (0.51, 0.75)***	0.57 (0.47, 0.7)***
Analgesic drugs except NSAIDs								
No	1	145	6.91	0	180	0.00	-	-
Yes	233	8977	25.95	200	12,280	16.29	0.62 (0.51, 0.75)***	0.58 (0.48, 0.7)***
Anti-lipid drug								
No	133	5240	25.38	105	7416	14.16	0.57 (0.44, 0.73)***	0.53 (0.41, 0.69)***
Yes	101	3882	26.02	95	5044	18.83	0.69 (0.52, 0.92)*	0.65 (0.49, 0.86)**
Steroid								
No	40	1663	24.06	28	2154	13.00	0.54 (0.33, 0.88)*	0.54 (0.33, 0.88)*
Yes	194	7460	26.01	172	10,306	16.69	0.63 (0.52, 0.78)***	0.59 (0.48, 0.73)***
Antiplatelet or anticoagulation agent								
No	63	3227	19.52	47	4305	10.92	0.55 (0.38, 0.81)**	0.51 (0.35, 0.75)***
Yes	171	5895	29.01	153	8155	18.76	0.64 (0.51, 0.79)***	0.61 (0.49, 0.76)***

CHM, Chinese herbal medicine; PY, person-years; IR, incidence rates, per 1,000 person-years; HR, hazard ratio; CI, confidence interval; COPD, chronic obstructive pulmonary disease; ARB, angiotensin receptor blocker; ACEI, angiotensin-converting enzyme inhibitors; NSAIDs, nonsteroidal anti-inflammatory drugs. *p < 0.05, **p < 0.01, ***p < 0.001.

Table 3 shows the results of the subgroup analysis according to the cumulative usage days of CHM among patients with CKD. Compared to the non-CHM group, there was a significant association between the reduced risk of IS and receiving CHM for more than 180 days (aHR = 0.51, 95% CI = 0.41–0.63).

**TABLE 3:** Hazard Ratios and 95% confidence intervals of ischemic stroke risk associated with the cumulative usage days of CHM during the follow-up period among patients with chronic kidney disease.

		Ischemic stroke			Crude HR (95%CI)	Adjusted HR (95%CI)
Number of drug days	Number	Event	PY	IR		
Non-CHM	3,149	234	9122	25.65		
CHM						
≤90 days	1,141	82	3961	20.70	0.76 (0.55, 1.06)	0.79 (0.62, 1.02)
91–180 days	439	33	1609	20.51	0.83 (0.60, 1.16)	0.75 (0.54, 1.05)
>180 days	1,569	85	6890	12.34	0.54 (0.43, 0.67)***	0.51 (0.41, 0.63)***

Crude HR represented relative hazard ratio; Adjusted HR represented adjusted hazard ratio mutually adjusted for age, gender, comorbidities and medication. PY, person-years; IR, incidence rates, per 1,000 person-years; HR, hazard ratio; CI, confidence interval; CHM, Chinese herbal medicine. ***p < 0.001.

The top three single herbs prescribed for CKD patients were *Salvia miltiorrhiza* Bunge [Lamiaceae; Salviae miltiorrhizae Radix et Rhizoma (Dan- Shen)], *Rheum palmatum* L [Polygonaceae; Rhei Radix et Rhizoma (Da-Huang)] and *Astragalus membranaceus* Bunge [Fabaceae; Astragali Radix (Huang-Qi)] and the three most commonly prescribed formulae were Ji-Sheng-Shen-Qi-Wan, Liu-Wei-Di-Huang-Wan, and Zhen-Wu-Tang (The information about composition and processing were shown in Supplementary Table S1). Further analysis using the proportional subdistribution hazards model of Fine and Gray revealed that Ji-Sheng-Shen-Qi-Wan, Liu-Wei-Di-Huang-Wan, and Zhen-Wu-Tang were significantly associated with a reduced risk of IS (Table 4).

**TABLE 4:** Hazard Ratios and 95% confidence intervals of ischemic stroke risk associated with the top three most commonly prescribed single herbs and formulas among patients with chronic kidney disease.

CHM prescription	Ischemic stroke		Hazard ratio (95%CI)	
	N	No. Of event	Crude	Adjusted†
Non-CHM users	3,149	234	1 (References)	1 (References)
CHM users				
Single herb				
*Salvia miltiorrhiza* Bunge (Dan- Shen)	273	39	0.6 (0.42, 0.84)**	0.60 (0.42, 0.85)**
*Rheum palmatum* L. (Da-Huang)	260	26	0.58 (0.39, 0.88)**	0.57 (0.38, 0.85)**
*Astragalus membranaceus* Bunge (Huang-Qi)	274	40	0.65 (0.45, 0.95)*	0.65 (0.44, 0.94)*
Formula				
Ji-Sheng-Shen-Qi-Wan	280	46	0.74 (0.54, 1.02)	0.70 (0.51, 0.97)*
Liu-Wei-Di-Huang-Wan	278	44	0.53 (0.38, 0.73)***	0.50 (0.36, 0.69)***
Zhen-Wu-Tang	268	34	0.60 (0.41, 0.86)**	0.57 (0.40, 0.82)**

CI, confidence interval; CHM, Chinese herbal medicine. †HR adjusted for age, gender, comorbidities, and medications. *p < 0.05, **p < 0.01, ***p < 0.001.

**FIGURE 3:**
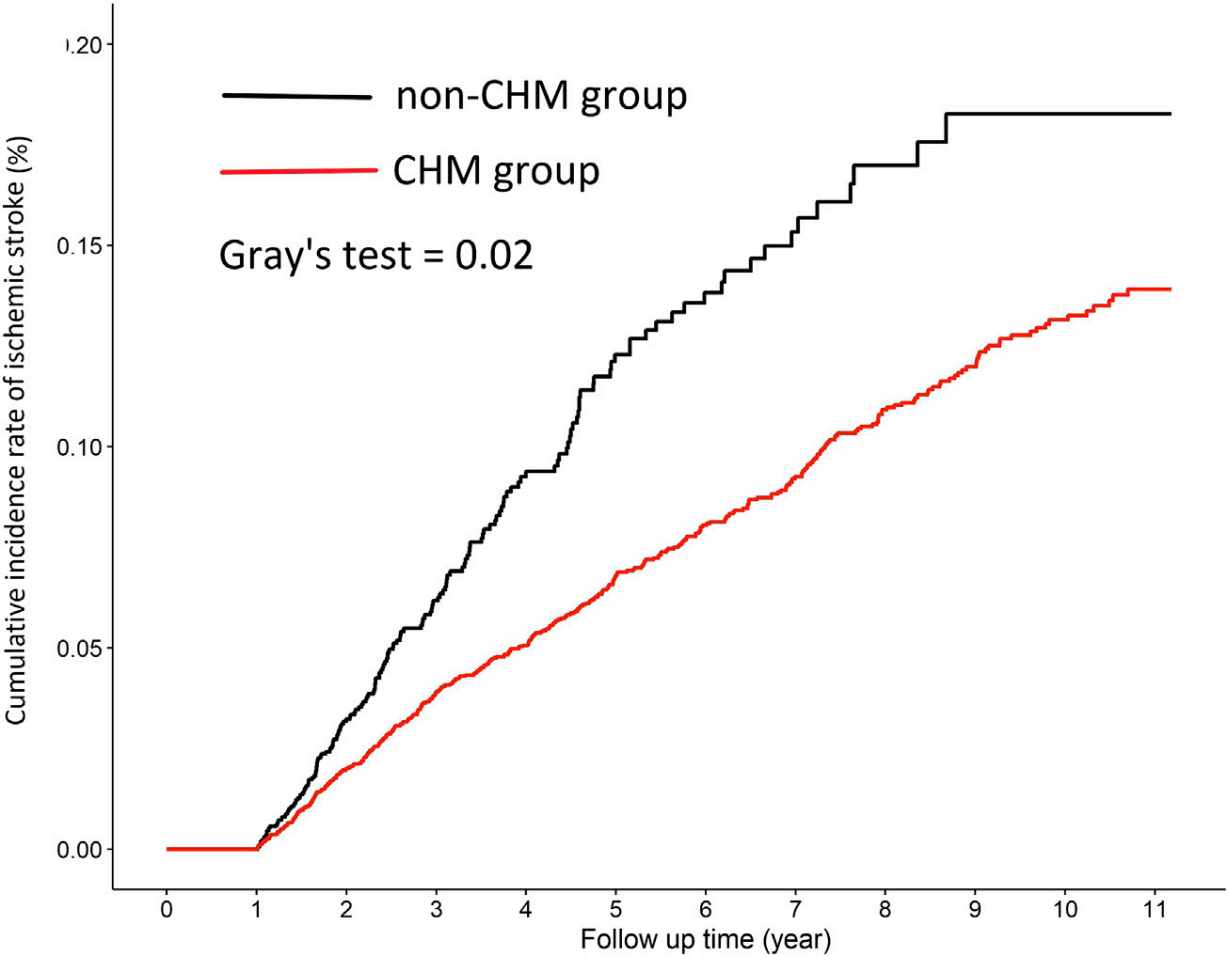
Cumulative incidence rate of ischemic stroke among patients with CKD in the CHM and non-CHM group (extending the period of case exclusion from 6 to 12 months).

In the sensitivity analysis of extending the period of case exclusion from 6 to 12 months, there were 2,808 patients in both the CHM group and non-CHM group after matching with propensity score at a ratio of 1:1 cohort sample by age (per 5 years-groups), gender, index year, comorbidities and medication. The basic characteristics in each group were similar with all standardized mean difference values < 0.1 (Table 5). The incidence rates of IS in the CHM and non-CHM group was 14.09 per 1000 person-years and 18.88 per 1000 person-years, respectively, and we found a 38% lower risk of IS in CHM group than in non-CHM group (aHR = 0.62, 95% CI: 0.50–0.77) (Table 6). In addition, both male and female patients with CKD receiving CHM as add-on therapy were significantly associated with a lower risk of IS [aHR (95% CI): 0.71 (0.54–0.94), 0.60 (0.41–0.87), respectively]. Moreover, Patients with CKD aged ≥65 years who received CHM had a significantly reduced IS risk compared with patients of the same age not receiving CHM (aHR: 0.66, 95% CI: 0.51–0.86). Figure 3 (extending the period of case exclusion from 6 to 12 months) displayed that the cumulative incidence rate of IS in the CHM group was significantly lower than that in the non-CHM group (Gray’s test, *p* = 0.02).

**TABLE 5:** Characteristics of patients with chronic kidney disease classified according to the use of Chinese herbal medicine after matching (extending the period of case exclusion from 6 months to 12 months).

	Non-CHM group(n = 2,808)		CHM group(n = 2,808)		Standardized
	n	%	N	%	
Difference					0.023
Gender					
Female	1015	36.1	984	35.0	
Male	1793	63.9	1824	65.0	
Age					
<50	419	14.9	327	11.6	0.097
50–64	791	28.2	885	31.5	0.073
≥65	1598	56.9	1596	56.8	0.001
mean (SD)	65.99	(15.1)	65.84	(13.0)	0.010
Comorbidity					
Hypertension	1435	51.1	1460	52.0	0.018
Diabetes mellitus	862	30.7	885	31.5	0.018
Hyperlipidemia	572	20.4	586	20.9	0.012
Atrial fibrillation	45	1.6	46	1.6	0.003
COPD	170	6.1	160	5.7	0.015
Chronic liver disease and cirrhosis	202	7.2	198	7.1	0.006
Coronary artery disease	62	2.2	63	2.2	0.002
Cancer	238	8.5	254	9.0	0.020
Medication					
Insulin Analogues	678	24.1	676	24.1	0.002
Oral hypoglycemic agent	994	35.4	1017	36.2	0.017
ARB/ACEI	1799	64.1	1856	66.1	0.043
Antihypertensive agent except ARB/ACEI	2595	92.4	2602	92.7	0.009
NSAIDs	2753	98.0	2758	98.2	0.013
Analgesic drugs except NSAIDs	2787	99.3	2784	99.1	0.012
Anti-lipid drug	1328	47.3	1376	49.0	0.034
Steroid	2385	84.9	2413	85.9	0.028
Antiplatelet or anticoagulation agent	1892	67.4	1922	68.4	0.023
Period from diagnosis date to index date, year [mean (SD)]	0.23	(0.26)	0.18	(0.25)	0.217
Follow-up period, year [mean (SD)]	3.21	(2.35)	3.94	(2.69)	0.292
Period from index date to ischemic stroke date, year [mean (SD)]	2.91	(1.67)	3.69	(2.23)	0.395

CKD, chronic kidney disease; CHM Chinese herbal medicine; SD standard deviation; COPD chronic obstructive pulmonary disease; ARB angiotensin receptor blocker; ACEI angiotensin-converting enzyme inhibitors; NSAIDs nonsteroidal anti-inflammatory drugs; A standardized mean difference of ≤0.10 indicates a negligible difference between the two cohorts.

**TABLE 6:** Incidence rates, hazard ratio and confidence intervals of ischemic stroke among chronic kidney disease patients with and without Chinese herbal medicine usage in the stratification of gender, age, comorbidities and medication (extending the period of case exclusion from 6 to 12 months).

Variables	Non-CHM group (n = 2,808)			CHM group (n = 2,808)			Crude HR (95%CI)	Adjusted HR (95%CI)
	Event	PY	Ir	Event	PY	Ir		
Overall								
Ischemic stroke	170	9006	18.88	156	11,075	14.09	0.67 (0.57, 0.83)***	0.62 (0.50, 0.77)***
Gender								
Female	67	3058	21.91	51	3876	13.16	0.55 (0.38, 0.80)**	0.60 (0.41, 0.87)**
Male	103	5947	17.32	105	7200	14.58	0.79 (0.60, 1.04)	0.71 (0.54, 0.94)*
Age								
≤49	8	1628	4.91	8	1544	5.18	1.05 (0.39, 2.80)	0.74 (0.24, 2.33)
50–64	35	2498	14.01	39	3619	10.78	0.68 (0.43, 1.07)	0.66 (0.42, 1.05)
≥65	127	4879	26.03	109	5912	18.44	0.66 (0.51, 0.85)**	0.66 (0.51, 0.86)**
Comorbidity								
Hypertension								
No	72	4696.34	15.33	65	5806	11.20	0.71 (0.50, 0.99)*	0.70 (0.50, 0.99)*
Yes	98	4309	22.74	91	5269	17.27	0.68 (0.51, 0.91)**	0.65 (0.48, 0.86)**
Diabetes mellitus								
No	112	6639	16.87	108	7895	13.68	0.78 (0.60, 1.01)	0.75 (0.57, 0.98)*
Yes	58	2367	24.51	48	3180	15.09	0.52 (0.35, 0.77)***	0.50 (0.34, 0.73)***
Hyperlipidemia								
No	145	7513	19.30	128	9067	14.12	0.70 (0.55, 0.88)**	0.67 (0.53, 0.86)**
Yes	25	1493	16.75	28	2009	13.94	0.68 (0.40, 1.18)	0.69 (0.39, 1.21)
Atrial fibrillation								
No	165	8909	18.52	151	10,937	13.81	0.69 (0.56, 0.87)**	0.67 (0.54, 0.84)***
Yes	5	97	51.67	5	139	36.04	0.72 (0.20, 2.51)	1.20 (0.29, 5.04)
COPD								
No	158	8501	18.59	145	10,451	13.87	0.70 (0.56, 0.87)**	0.67 (0.53, 0.84)***
Yes	12	505	23.77	11	624	17.61	0.66 (0.28, 1.52)	0.65 (0.26, 1.59)
Chronic liver disease and cirrhosis								
No	157	8351	18.80	146	10,325	14.14	0.70 (0.56, 0.87)**	0.68 (0.54, 0.85)***
Yes	13	654	19.88	10	750	13.33	0.67 (0.29, 1.53)	0.60 (0.24, 1.45)
Chronic artery disease								
No	160	8840	18.10	153	10,809	14.16	0.73 (0.58, 0.91)**	0.70 (0.56, 0.88)**
Yes	10	166	60.26	3	267	11.26	0.14 (0.04, 0.55)**	0.16 (0.03, 0.85)*
Cancer								
No	155	8326	18.62	144	10,180	14.15	0.71 (0.56, 0.89)**	0.69 (0.55, 0.86)**
Yes	15	679	22.09	12	896	13.40	0.56 (0.26, 1.20)	0.49 (0.22, 1.08)
Medications								
Insulin Analogues								
No	116	7059	16.43	109	8632	12.63	0.73 (0.56, 0.94)*	0.69 (0.53, 0.90)**
Yes	54	1947	27.74	47	2444	19.23	0.60 (0.41, 0.89)*	0.58 (0.39, 0.87)**
Oral hypoglycemic agent								
No	106	6211	17.07	93	7425	12.53	0.70 (0.53, 0.93)*	0.66 (0.50, 0.87)**
Yes	64	2795	22.90	63	3650	17.26	0.65 (0.46, 0.92)*	0.65 (0.45, 0.92)*
ARB/ACEI								
No	47	3446	13.64	40	4041	9.90	0.69 (0.45, 1.05)	0.72 (0.47, 1.11)
Yes	123	5559	22.13	116	7035	16.49	0.68 (0.53, 0.88)**	0.66 (0.51, 0.86)**
Antihypertensive agent except ARB/ACEI								
No	5	891	5.61	5	1053	4.75	0.78 (0.22, 2.69)	0.40 (0.08, 1.93)
Yes	165	8115	20.33	151	10,022	15.07	0.69 (0.55, 0.86)***	0.67 (0.53, 0.83)***
NSAID								
No	3	251	11.95	3	299	10.05	0.70 (0.14, 3.49)	0.72 (0.30, 2.59)
Yes	167	8755	19.08	153	10,777	14.20	0.69 (0.56, 0.87)**	0.67 (0.54, 0.83)***
Analgesic drugs except NSAIDs								
No	0	146	0.00	0	162	0.00	0.69 (0.56, 0.86)***	0.67 (0.54, 0.83)***
Yes	170	8860	19.19	156	10,913	14.30		
Anti-lipid drug								
No	93	5141	18.09	83	6115	13.57	0.72 (0.53, 0.97)*	0.69 (0.51, 0.93)*
Yes	77	3864	19.93	73	4961	14.72	0.66 (0.48, 0.91)*	0.65 (0.47, 0.90)**
Steroid								
No	29	1660	17.47	33	1929	17.11	0.90 (0.55, 1.49)	0.95 (0.57, 1.60)
Yes	141	7346	19.19	123	9146	13.45	0.66 (0.51, 0.84)***	0.61 (0.48, 0.78)***
Antiplatelet or anticoagulation agent								
No	42	3213	13.07	40	3821	10.47	0.74 (0.48, 1.14)	0.72 (0.47, 1.13)
Yes	128	5793	22.10	116	7254	15.99	0.67 (0.52, 0.86)**	0.66 (0.51, 0.85)**

CHM, Chinese herbal medicine; PY, person-years; IR, incidence rates, per 1,000 person-years; HR, hazard ratio; CI, confidence interval; COPD, chronic obstructive pulmonary disease; ARB, angiotensin receptor blocker; ACEI, angiotensin- converting enzyme inhibitors; NSAIDs, nonsteroidal anti-inflammatory drugs. *p < 0.05, **p < 0.01, ***p < 0.001

## Discussion

This nationwide cohort study using real-world data revealed that the use of CHM as add-on therapy was associated with a lower risk of IS among patients with CKD. In the subgroup analysis of different cumulative CHM usage days, we identified an association between longer CHM usage (>180 days) and a reduced risk of IS. To date, few studies have examined conventional medicine along with CHM as adjunctive therapy in relation to the risk of IS among patients with CKD. This study used a large-scale nationwide cohort to address this knowledge gap.

Although the precise mechanism was not clear, multiple factors may contribute to the development of IS among CKD patients. The possible explanation might be that the commonly prescribed CHM attenuates inflammation response and oxidative stress to block the arteriosclerosis progression. Liu-Wei-Di-Huang-Wan was shown to prevent the progression of renal fibrosis by inhibiting the NF-κB pathway in rats with diabetic nephropathy (Xu et al., 2017). Moreover, Zhen-Wu-Tang was demonstrated to have a protective effect against renal fibrosis by alleviating oxidative stress. After treatment with Zhen-Wu-Tang, reactive oxygen species decreased in the kidney and peroxisome proliferator-activated receptor gamma (PPARγ) was up-regulated. PPARγ had anti-inflammatory and anti-fibrosis effects by inhibiting the expression of transforming growth factor beta 1 (TGF-β1) and NF-κB, an important factor in the atherosclerotic process (Li et al., 2018). *Astragalus membranaceus* Bunge [Fabaceae; Astragali Radix (Huang-Qi)] and *Salvia miltiorrhiza* Bunge [Lamiaceae; Salviae miltiorrhizae Radix et Rhizoma (Dan-Shen)], the most commonly prescribed single herbs, have also been reported to attenuate kidney injury via anti-inflammatory effects by suppressing the activation of NF-κB signaling pathways in the animal model (Cai et al., 2018; Du et al., 2018). *Rheum palmatum* L [Polygonaceae; Rhei Radix et Rhizoma (Da-Huang)] has been shown to have effects of attenuating renal inflammatory injury through decreasing the level of certain proinflammatory cytokines, including prostaglandin E, tumor necrosis factor-α, and TGF-β1 (Meng et al., 2015; Hu et al., 2020). The commonly used CHM, via mitigating inflammation response and oxidative stress, was associated with a decreased risk of IS among patients with CKD.

**FIGURE 4:**
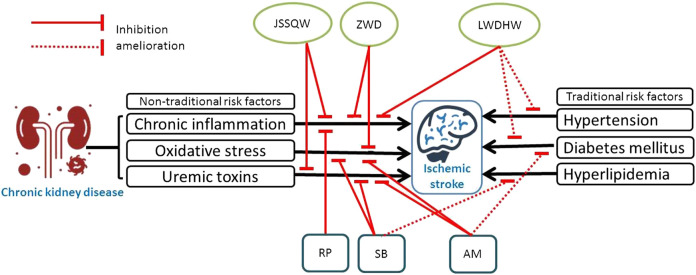
Schematic diagram illustrating the proposed mechanisms for the benefit of commonly prescribed CHM in reducing the risk of ischemic stroke.

Besides suppressing inflammation and oxidative stress leading to vascular fibrosis or arteriosclerosis, some commonly prescribe CHM has been demonstrated to prevent indoxyl sulfate (a uremic toxin)-induced endothelial dysfunction. For examples, previous investigators (Yamabe et al., 2005) have demonstrated that Ji-Sheng-Shen-Qi-Wan could decrease the level of uremic toxins and hydroxyl radicals to ameliorate renal damage and failure in a subtotal nephrectomy rat model. Ji-Sheng-Shen-Qi-Wan also could reduce interstitial fibrosis and inflammation significantly (Yamabe et al., 2005). Moreover, Astragaloside IV, one of the major compounds of *Astragalus membranaceus* Bunge [Fabaceae; Astragali Radix (Huang-Qi)]*,* was shown to alleviate indoxyl sulfate-induced kidney injury through suppressing oxidative stress response (Ji et al., 2018). Salvianolic acids, the most effective and abundant compounds extracted from *Salvia miltiorrhiza* Bunge [Lamiaceae; Salviae miltiorrhizae Radix et Rhizoma (Dan-Shen)] could significantly enhance the dialysis removal of protein-bound indoxyl sulfate (Li et al., 2019). In addition, *Salvia miltiorrhiza* Bunge [Lamiaceae; Salviae miltiorrhizae Radix et Rhizoma (Dan-Shen)] has also been found to inhibit the elevation of indoxyl sulfate, which is thought to accelerate atherogenesis in patients with CKD (Li et al., 2019; Nakano et al., 2019). Furthermore, over 50% of patients with CKD are known to have multiple comorbidities including hypertension, diabetes mellitus, and hyperlipidemia that may negatively impact IS (Kelly et al., 2021). Certain commonly prescribed CHM, such as Liu-Wei-Di-Huang-Wan, *Salvia miltiorrhiza* Bunge [Lamiaceae; Salviae miltiorrhizae Radix et Rhizoma (Dan-Shen)] and *Astragalus membranaceus* Bunge [Fabaceae; Astragali Radix (Huang-Qi)] have been shown to confer benefits in ameliorating these comorbidities (Wang J. et al., 2012; Agyemang et al., 2013; Wang et al., 2017; Gao et al., 2018) thus contributing to the reduction in IS risk. Accordingly, this might be the possible reason that CKD patients comorbid with traditional risk factors of IS taking CHM have a greatly reduced risk of IS than those not taking CHM. Collectively, certain formulae and single herbs taken by patients with CKD might exert anti-inflammatory effects, inhibit oxidative stress response, reduce damages by uremic toxins, and alleviate comorbidity to mitigate the risk of IS. The proposed mechanisms for the benefit of commonly used CHM are described in Figure 4.

Another major finding in our study was that the use of CHM for more than 180 days was associated with a lower risk of IS in the CHM group. It seems possible that CHM required at least 180 days to achieve an adequate protective effect in patients with CKD. Although no firm evidence indicated the minimum cumulative usage days for renal protection in CHM, the minimum effective usage days of CHM in our study was similar to the treatment duration of certain randomized controlled trials. Patients with primary glomerular disease, the most common type of CKD, receiving *Abelmoschus manihot* (L.) Medik [Malvaceae; Abelmoschi Corolla (Huang-Shu-Kui)] combined with losartan for 24 weeks showed a more reduction in 24-hour proteinuria than those taking losartan alone (Zhang et al., 2014). In addition, compared with taking benazepril alone, patients with stage 3 CKD taking CHM combined with benazepril for 24 weeks had lower 24-hour proteinuria levels and higher eGFR values (Wang Y. J. et al., 2012). A previous study confirmed that decreased eGFR was one of the main risk factors of IS (Synhaeve et al., 2016). In brief, CKD is characterized by increased oxidative stress and chronic systemic inflammation, and taking CHM as add-on therapy for >180 days might exert an adequate anti-inflammatory and anti-oxidative effect to reduce the risk of IS among CKD patients. Moreover, our analysis reveals that the CHM-related reduction in IS risk was more apparent in CKD patients aged ≥65 years compared to other age groups. The possible reason for this observation is that CKD patients aged ≥65 years are known to have many traditional risk factors of IS that could substantially elevate the risk of IS. (Peng et al., 2011; Liang et al., 2020; Tsao et al., 2021). CHM may exert a potential effect in ameliorating the impact of comorbidities (Wang J. et al., 2012; Agyemang et al., 2013; Wang et al., 2017; Gao et al., 2018) thus conducing to the decreased risk of IS in this age group.

The Yin-Yang theory of traditional Chinese medicine may explain why clinical doctors choose those herbs as CKD treatments. CKD is characterized by three syndromes based on the theory of Chinese medicine, syndrome of Yang deficincy and water overflowing, syndrome of Qi deficiency and blood stasis, and syndrome of kidney Yin deficincy (Wang et al., 2016). The clinical symptom of Yang deficiency and water overflowing in CKD patients is swelling over lower extremities. The commonly prescribed formulae such as Ji-Sheng-Shen-Qi-Wan and Zhen-Wu-Tang could exert the function of activating Yang and promoting diuresis to alleviate the swelling. In addition, the typical feature of Qi deficiency and blood stasis in CKD patients is fatigue. Certain single herbs including *Salvia miltiorrhiza* Bunge [Lamiaceae; Salviae miltiorrhizae Radix et Rhizoma (Dan-Shen)], *Rheum palmatum* L [Polygonaceae; Rhei Radix et Rhizoma (Da-Huang)] and *Astragalus membranaceus* Bunge [Fabaceae; Astragali Radix (Huang-Qi)] have the potential to enrich Qi and remove blood stasis to invigorate these patients. Furthermore, the clinical signs of kidney Yin deficiency in CKD patients are dizziness, tinnitus, and oliguria. The second most commonly prescribed formula, Liu-Wei-Di-Huang-Wan, could nourish kidney Yin to ameliorate the associated signs. Our findings provide evidence suggesting that those herbs may lower the IS risk in CKD patients, a benefit that is beyond the relief of clinical symptoms.

The strength of our study was using large-scale real-world data with longitudinal follow-up. Compared to randomized clinical trials, real world studies had broader generalizability and give evidence of effectiveness in daily practice settings (Blonde et al., 2018). The study also had limitations which may have affected results. First, important clinical characteristics, such as smoking, body mass index, diet, exercise habits, and eGFR were unavailable from our database. However, we applied the proportional subdistribution hazards model of Fine and Gray by controlling for the available potential confounding factors of IS, including age, gender, comorbidities, and medications, to make the two groups comparable. Second, there was a possibility of misclassification bias in the disease coding. To reduce the bias, we identified patients with CKD through at least three outpatient visits or one inpatient visit within 1 year, and patients who were diagnosed with IS through at least three outpatient visits or one inpatient visit. Furthermore, the accuracy of the NHIRD for patients with a diagnosis of IS and CKD is high, and the database we used is a valid resource for this research (Cheng et al., 2011; Lin et al., 2015). Third, medications compliance should be noted. We supposed that all of the CHM prescribed was taken by the patients, but the actual dosage taken might have been overestimated due to poor compliance. The compliance is likely to be decreased over time especially in the case of a long follow-up period. Poor compliance may be associated with a higher risk of IS among patients with CKD, thus supporting the robustness of our findings. Last, the present study investigated patients with CKD in Taiwan; thus, the application of the study findings to populations of other countries or races is inadequate. Forth, we noted that the IS risk in female patients with CKD who used CHM was much lower than that in male patients with CKD who used CHM. The exact reasons for this difference remain unclear, but it is known that many physiologic and pathophysiologic aspects of females and males are different.

## Conclusion

In this real-world, nationwide cohort study, the use of CHM as add-on therapy for patients with CKD was associated with a decreased risk of IS compared to using conventional treatment alone. In particular, CKD patients taking CHM for >180 days had a significantly lower risk of IS. Additionally, Ji-Sheng-Shen-Qi-Wan, Liu-Wei-Di-Huang-Wan, and Zhen-Wu-Tang might be associated with a 30%–50% reduced risk of IS. Although adjunctive CHM may have beneficial effects for IS prevention in patients with CKD, further randomized clinical trials are required to clarify the causal relationship of these results. Also, patients with other chronic inflammatory diseases such as rheumatoid arthritis (Tam et al., 2018; Tang et al., 2019) also have a high risk of IS. Whether adjunctive CHM may have beneficial effects for IS prevention in these patients warrant further investigation.

## Data Availability

The datasets presented in this article are not readily available because the datasets is limited to research purposes only. The datasets we analyzed from NHIRD was provided by the National Health Insurance Administration and maintained by the National Health Research Institutes of Taiwan. The use of NHIRD is limited to research purposes only. Applicants must follow the Computer-Processed Personal Data Protection Law and related regulations of National Health Insurance Administration and National Health Research Institutes. Requests to access the datasets should be directed to National Health Insurance Administration, Ministry of Health and Welfare, Taiwan, nhird@nhri.edu.tw.
