# Supplementary material for: Lower risk of ischemic stroke among patients with chronic kidney disease using chinese herbal medicine as add-on therapy: A real-world nationwide cohort study
Source: Front Pharmacol. 2022 Aug 11;13:883148. doi: 10.3389/fphar.2022.883148 (PMC9403506; doi:10.3389/fphar.2022.883148)
Supplement: Supplementary file 1 [file Table1.pdf]

Supplementary Table 1. The information about composition and processing of the three most commonly prescribed formulae

Name	Ingredients	Percentage (%)	Processing
Ji-Sheng-Shen-Qi-Wan	*Rehmannia glutinosa* (Gaertn.) DC. [Orobanchaceae; Rehmanniae Radix (Shou-Di-Huang)]	26.0%	These three formulae were manufactured by Good Manufacturing Practice (GMP) pharmaceutical companies in Taiwan according to the regulation of the Department of Chinese Medicine and Pharmacy, Ministry of Health and Welfare, Taiwan. The processing of the formula starts from boiling certain single herbs in water for the decoction, and the decoction was condensed to a concentrated solution for spray drying. Then, add a proper amount of starch to make granulations.
	Cornus officinalis Siebold & Zucc. [Cornaceae; Corni Sarcocarpium (Shan-Zhu-Yu)]	13.0%	
	Dioscorea oppositifolia L. [Dioscoreaceae; Dioscoreae Rhizoma (Shan-Yao)]	13.0%	
	Wolfiporia cocos (Schw.) Ryv. & Cilbn [Polyporaceae; Poria (Fu-Ling)]	10.0%	
	Paeonia suffruticosa Andrews [Paeoniaceae; Moutan Radicis Cortex (Mu-Dan-Pi)]	10.0%	
	Alisma orientalis (Sam.) Juzep. [Alismataceae; Alismatis Rhizoma (Ze-Xie)]	10.0%	
	Aconitum carmichaeli Debeaux [Ranunculaceae; Aconiti Lateralis Radix Praeparata (Pao-Fu-Zi)]	3.0%	
	Cinnamomum cassia Presl, [Lauraceae; Cinnamomi Cortex (Rou-Gui)]	3.0%	
	Achyranthes bidentata Blume [Amaranthaceae; Achyranthis bidentatae Radix (Huai-Niu-Xi)]	6.0%	
	Plantago asiatica L. [Plantaginaceae; Plantaginis Semen (Che-Qian-Zi)]	6.0%	
Liu-Wei-Di-Huang-Wan	*Rehmannia glutinosa* (Gaertn.) DC. [Orobanchaceae; Rehmanniae Radix (Shou-Di-Huang)]	32.0%	
	Cornus officinalis Siebold & Zucc. [Cornaceae; Corni Sarcocarpium (Shan-Zhu-Yu)]	16.0%	
	Dioscorea oppositifolia L. [Dioscoreaceae; Dioscoreae Rhizoma (Shan-Yao)]	16.0%	

	Wolfiporia cocos (Schw.) Ryv. & Cilbn [Polyporaceae; Poria (Fu-Ling)]	12.0%
	Paeonia suffruticosa Andrews [Paeoniaceae; Moutan Radicis Cortex (Mu-Dan-Pi)]	12.0%
	Alisma orientalis (Sam.) Juzep. [Alismataceae; Alismatis Rhizoma (Ze-Xie)]	12.0%
Zhen-Wu-Tang	*Wolfiporia cocos* (Schw.) Ryv. & Cilbn [Polyporaceae; Poria (Fu-Ling)]	25.0%
	Paeonia lactiflora Pall. [Paeoniaceae; Paeoniae Alba Radix (Bai-Shao-Yao)]	25.0%
	Zingiber officinale Roscoe [Zingiberaceae; Zingiberis Rhizoma Recens (Sheng-Jiang)]	25.0%
	*Atractylodes macrocephal*a Koidz. [Asteraceae; Atractylodis macrocephalae Rhizoma (Bai-Zhu)]	16.5%
	Aconitum carmichaeli Debeaux [Ranunculaceae; Aconiti Lateralis Radix Praeparata (Pao-Fu-Zi)]	8.5%
